# Neoadjuvant oxaliplatin and capecitabine combined with bevacizumab plus radiotherapy for locally advanced rectal cancer: results of a single-institute phase II study

**DOI:** 10.1186/s40880-018-0294-z

**Published:** 2018-05-21

**Authors:** Xin Yu, Qiao-xuan Wang, Wei-wei Xiao, Hui Chang, Zhi-fan Zeng, Zhen-hai Lu, Xiao-jun Wu, Gong Chen, Zhi-zhong Pan, De-sen Wan, Pei-rong Ding, Yuan-hong Gao

**Affiliations:** 1 Sun Yat-sen University Cancer Center; State Key Laboratory of Oncology in South China; Collaborative Innovation Center for Cancer Medicine, Guangzhou, P. R. China; 2 Department of Radiation Oncology, Sun Yat-sen University Cancer Center, 651 Dongfeng Rd East, Guangzhou, 510060 P. R. China; 3 Department of Colorectal Surgery, Sun Yat-sen University Cancer Center, 651 Dongfeng Rd East, Guangzhou, 510060 P. R. China

**Keywords:** Bevacizumab, Neoadjuvant chemoradiotherapy, Locally advanced rectal cancer, Safety, Efficacy

## Abstract

**Background:**

Neoadjuvant chemoradiotherapy followed by surgery is recommended as the standard of care for locally advanced rectal cancer, reducing local recurrence but not distant metastasis. Intensified systemic therapy is warranted to reduce the risk of distant metastasis. The present study aimed to evaluate the safety and efficacy of neoadjuvant oxaliplatin and capecitabine (XELOX) combined with bevacizumab plus radiotherapy for locally advanced rectal cancer.

**Methods:**

Patients with stages II to III rectal cancer received one cycle of induction chemotherapy and concurrent chemoradiotherapy with XELOX plus bevacizumab. Surgery was performed 6–8 weeks after completion of radiotherapy, and postoperative chemotherapy with three cycles of XELOX and two cycles of capecitabine were given. The primary endpoints were pathologic complete response (pCR) rate and safety, and the secondary endpoints were 3-year overall survival and progression-free survival.

**Results:**

Forty-five patients were enrolled between February 2013 and April 2015. All completed the neoadjuvant therapy. Seven patients (15.6%) refused subsequent surgical therapy for personal reasons, and the other 38 patients received radical resection, with a sphincter preservation rate of 84.2% and a pCR rate of 39.5%. Toxicity was acceptable, with grades 3–4 hematological toxicity and diarrhea observed in six and two patients, respectively. Incidence of anastomotic leak that required surgical intervention was 13.3%. After a median follow-up period of 37 months, five patients developed disease progression and two died of cancer. The 3-year overall survival rate and 3-year progression-free survival rate were 95.3% and 88.6%, respectively.

**Conclusions:**

The addition of bevacizumab to neoadjuvant chemoradiotherapy resulted in a satisfying pCR rate and 3-year survival, but also may increase the risk of anastomotic leak, thus this regimen is not suitable to be considered for regular recommendation for locally advanced rectal cancer.

*Trial registration* Clinicaltrials.govidentifierNCT01818973

## Background

Colorectal cancer has now become the fourth common cancer in China and the fifth leading cause of cancer death [1], with many patients diagnosed at advanced stages, bringing a heavy social and economic burden. Until the 1980s, patients with locally advanced rectal cancer (cT3–4 and/or cN+) had high incidences of both local recurrence and distant metastasis. To solve this problem, total mesorectal excision (TME) instead of conventional surgery was developed, which improved outcomes in locoregional control and survival in rectal cancer [2, 3]. Meanwhile, neoadjuvant chemoradiation (neoCRT) was also widely investigated. It was demonstrated that neoCRT significantly reduced pelvic recurrences and increased sphincter-sparing surgery rates, but had no clear improvement on survival [4, 5]. According to the National Comprehensive Cancer Network (NCCN) guidelines [6], neoCRT followed by TME is now recommended as the standard of care for locally advanced rectal cancer.

While locoregional recurrence rates have been reduced to only 4%–8%, 5-year disease-free survival rates in locally advanced rectal cancer remain low at 59%–77% [4, 5, 7], indicating insufficient control over systemic failure. In prevalent treatment schedules, systemic chemotherapy is put in the adjuvant setting after surgery, which means that patients have to receive systemic therapy in about 3–4 months after the diagnosis of cancer; this may account for systemic failure, especially for patients who have already had micrometastases in the initial diagnosis. To address this problem, more effective systemic treatment regimens are needed.

Recently, a series of studies demonstrated that patients with pathological complete response (pCR) after chemoradiotherapy achieved a good prognosis compared with those who had pathological residual disease, in terms of disease-free survival (DFS) and overall survival (OS) [8, 9]. Therefore, some clinical trials were designed to increase pCR by adding new anti-cancer drugs to neoCRT. Oxaliplatin plus capecitabine (the XELOX regimen) has been recommended as an option of induction therapy [10]. The addition of molecular-targeted therapy to increase the rate of responders and to address micrometastases has also been discussed [11].

Bevacizumab, a monoclonal antibody that blocks vascular endothelial growth factor (VEGF), is a critical mediator of tumor angiogenesis [12]. It has been demonstrated that bevacizumab significantly improves OS in patients with metastatic colorectal cancer [13, 14]. Moreover, anti-VEGF antibody has been reported to compensate for the resistance to radiation and augment tumor response in preclinical models [15, 16]. However, the use of bevacizumab as neoadjuvant treatment for locally advanced rectal cancer remains unclear. Several phase II studies have been conducted using bevacizumab in combination with chemoradiotherapy, demonstrating its feasibility with acceptable toxicity [17–19], but none were conducted in Asian populations.

In our institution, we began the use of XELOX as the concurrent chemotherapy regimen in 2007. In our preliminary studies, we demonstrated that induction chemotherapy followed by chemoradiotherapy with the XELOX regimen was well tolerated and brought about a satisfactory pCR in high-risk locally advanced rectal cancer [20–22]. Here we designed a single center pilot study to determine whether adding bevacizumab to neoadjuvant treatment with oxaliplatin and capecitabine plus radiotherapy was a safe and effective strategy for clinical stages II to III rectal cancer.

## Paitents and methods

### Study design

The present study was a single-arm, single-center, open-label phase II trial conducted at Sun Yat-sen University Cancer Center. The research hypothesis was that bevacizumab combined with the XELOX regimen plus radiotherapy could increase the pCR rate in locally advanced rectal cancer with acceptable toxicity. The primary endpoint was pCR rate at surgery. According to previous trials, pCR rate for patients with locally advanced rectal cancer treated with radiation and capecitabine is approximately 13%, and the estimated pCR rate of our trials is estimated to be about 40%. With an α-error of 0.05 and a β-error of 0.20, 41 patients were required. Considering that 10% of patients were likely to be non-evaluable, we planned to enroll 45 patients in this trial. Our primary endpoint also included safety, which was measured by the incidence of adverse events. Secondary end-points were 3-year OS and progression-free survival (PFS).

### Patient selection

Eligibility criteria included histopathologically confirmed rectal adenocarcinoma with clinical stages II (T3–4N0M0) or III (T1–4N1–2M0), located within 12 cm of the anal verge; an age of 18–70 years with a life expectancy greater than 2 years; an Eastern Cooperative Oncology Group performance status of 0 or 1; an adequate hematologic, renal, and hepatic function (i.e., neutrophils ≥ 1.5 × 10^9^/L; hemoglobin ≥ 9 g/dL; platelet count ≥ 100 × 10^9^/L; total bilirubin < 1.5 mg/dL, and serum glutamic–oxaloacetic transaminase ≤ 2 times the upper limit of normal).

Patients were excluded if they had significant cardiovascular disease, including poorly controlled hypertension, unstable angina, myocardial infarction or stroke, or heavy proteinuria or thrombotic episode within 6 months prior to enrollment. Patients were also excluded if they had had previous treatment including surgery (except for enterostomy), chemotherapy, radiotherapy, biotherapy, or targeted therapy for rectal cancer, or had had another primary cancer within 5 years prior to enrollment.

### Ethics

The present study was approved by Sun Yat-sen University Cancer Center Institutional Review Board on Medical Ethics, and registered at ClinicalTrials.gov (NCT 01818973). All study participants provided written informed consent before treatment.

### Treatment schedule

Bevacizumab was supplied by Roche (Shanghai, China) in commercially available formulations. The treatment schema was as follows. Patients received one cycle of chemotherapy that consisted of intravenous bevacizumab (7.5 mg/kg on day 1) plus XELOX (intravenous oxaliplatin 130 mg/m^2^ on day 1, with oral capecitabine 1000 mg/m^2^ twice daily on days 1–15 of the cycle) 3 weeks before concurrent CRT.

Radiation treatment consisted of 50 Gy radiation to the primary tumor and positive lymph nodes, and 45 Gy to regional lymphatic drainage including the mesorectal, presacral, and internal iliac lymph nodes up to the level of the bottom part of the fifth lumbar vertebra, delivered with 6 MV photons through a volumetric modulated arc therapy (VMAT) technique, in 25 fractions over 5 weeks. During the course of radiotherapy, two cycles of bevacizumab (7.5 mg/kg, on days 22 and 43) and modified XELOX (oxaliplatin 100 mg/m^2^ on days 22 and 43, and capecitabine 1000 mg/m^2^, on days 22–36 and day 43–57) were given synchronously.

Surgical resection with TME was carried out 6–8 weeks after completion of neoadjuvant treatment. Creation of a temporary diverting ostomy was at the discretion of the primary surgeon during radical resection. After recovery from surgery, three cycles of XELOX and two cycles of capecitabine were recommended.

### Treatment evaluation and follow up

Pretreatment evaluation included a complete medical history, physical examination, hematology, liver and kidney function tests, coagulation profile, electrocardiogram, and colonoscopy with biopsy. Carcinoembryonic antigen (CEA) and carbohydrate antigen 19-9 (CA19-9) levels were measured. Endorectal ultrasound (ERUS) associated with enhanced pelvic magnetic resonance imaging (MRI) or computed tomography (CT) scans were performed to estimate tumor size, along with chest and abdomen CT scans to rule out metastatic disease. Immediately before surgery, pelvic MRI, chest and abdominal CT, and CEA and CA199 measurements were repeated. Patients were staged according to the 2010 International Union against Cancer [UICC] staging system [23]. Surgical evaluation was undertaken by the primary surgeon at baseline and before surgery.

Based on Response Evaluation Criteria in Solid Tumors (RECIST, Version 1.1) [24], clinical evaluation of tumor response was classified into four levels: complete response (CR), partial response (PR), stable disease (SD) and progressive disease (PD).

The pathologic examination of surgical specimens was performed according to the American Joint Committee on Cancer (AJCC, Version 7) [18]. R0 resection was defined as complete tumor resection with all margins histopathologically negative. A four-point tumor regression grading (TRG) modified from Ryan et al. was used to categorize the tumor shrinkage levels after preoperative therapy: (1) grade 0, also defined as pCR, complete response with no viable cancer cells; (2) grade 1, also defined as near pathological complete response (near-pCR) [25], only small clusters or single cancer cells remaining; (3) grade 2, residual cancer remaining, but with predominant fibrosis; and (4) grade 3, poor response with extensive residual cancer [23, 26]. Down-staging was defined as lower ypT compared with the pretreatment clinical T, or lower ypN compared with the pretreatment clinical N.

Toxicities were evaluated according the National Cancer Institute Common Toxicity Criteria 3.0 (NCI-CTC 3.0) [27], and postoperative complications were evaluated at the post-surgery visit after all treatment was completed.

All patients were followed at 3-month intervals during the first 2 years after surgery and at least every 6 months thereafter for an additional period of 3 years. Local recurrence was defined as a clinically proven relapse anywhere within the pelvis. Distant metastasis was any tumor dissemination outside the pelvis including peritoneal carcinomatosis that occurred during follow-up. PFS was defined as the time from the date of trial entry until disease progression, relapse, or death from any cause. OS was calculated from the date of trial entry until death from any cause or was censored at last follow-up.

### Statistical analysis

Characteristics were described in terms of frequency for the categorical variables and medians for non-normally distributed data. OS and PFS were calculated using the Kaplan–Meier method. Proportions were compared using a χ^2^ test. All statistical tests were two-sided. Significance was set at *P* < 0.05. Statistical analyses were performed using the Statistical Package for the Social Sciences Program (SPSS Inc. Chicago, IL, Version 19.0 for Windows).

## Results

### Baseline characteristics

Forty-five patients were enrolled between March 2013 and April 2015, including 25 (55.6%) males and 20 (44.4%) females, with a median age of 48 years (range 16–69 years). Local staging was performed with MRI of the pelvis for 42 (93.3%) of the 45 eligible patients, and with endorectal ultrasound plus pelvic CT scan for the other 3 (6.7%) patients. The majority (40/45, 88.9%) had clinically node-positive tumors, and more than half of the patients (26/45, 57.8%) were cT4 (Table 1).

**Table 1:** Baseline characteristics of 45 patients with locally advanced rectal cancer enrolled in the present study

Characteristic	No. of patients (%)
Age (years)	
≤ 60	37 (82.2)
> 60	8 (17.8)
Gender	
Male	25 (55.6)
Female	20 (44.4)
CEA (mg/mL)	
< 5.00	28 (62.2)
≥ 5.00	17 (37.8)
CA19-9 (μg/mL)	
< 27	31 (68.9)
≥ 27	14 (31.1)
Clinical T category	
T2	1 (2.2)
T3	18 (40.0)
T4a	21 (46.7)
T4b	5 (11.1)
Clinical N category	
N0	5 (11.1)
N1	15 (33.3)
N2	25 (55.6)
Clinical disease category	
Stage II	5 (11.1)
Stage III	
IIIA	1 (2.2)
IIIB	12 (26.7)
IIIC	27 (60)
Location from anal verge (cm)	
0–5	27 (60.0)
> 5–10	15 (33.3)
> 10	3 (6.7)
Tumor differentiation	
Well differentiated (G1)	2 (4.4)
Moderately differentiated (G2)	28 (62.2)
Poor differentiated (G3)	5 (11.1)
Other or missing	10 (22.2)

### Toxicities

Toxicities (Table 2) were mainly grades 1–2, and grades 3–4 events occurred in nine of 45 (20.0%) patients. Six (13.3%) patients had grades 3–4 hematologic toxicity (leukopenia and neutropenia), and three (6.7%) patients had grade 3 non-hematologic toxicity, including two cases (4.4%) of diarrhea and one case (2.2%) of radiation dermatitis. No severe bevacizumab-related toxic effects including hypertension, bleeding, thromboembolism or gastric-intestinal perforation were observed, but bevacizumab-related proteinuria occurred in two cases. With the exception of two patients, all individuals with toxic events recovered after supportive care without significant interruptions to treatment. One patient who had grade 3 diarrhea and dehydration after his first cycle of XELOX plus bevacizumab continued treatment with FOLFOX plus bevacizumab without difficulty, and another who suffered from grade 3 mucositis after her second cycle of XELOX plus bevacizumab continued with neoadjuvant radiotherapy alone. The remaining 43 of 45 (95.6%) patients completed three cycles of XELOX plus bevacizumab.

**Table 2:** Summary of acute adverse effects in 45 patients treated with bevacizumab in combination with neoadjuvant chemoradiotherapy

Toxicity (NCI-CTC Version 3.0)	No. of patients			
	Grade 1	Grade 2	Grade 3	Grade 4
Hematological				
Leukopenia	11	8	3	1
Neutropenia	16	6	1	1
Thrombocytopenia	1	0	0	0
Non-hematological				
Diarrhea	10	2	2	0
Nausea or vomiting	17	2	0	0
Hand-foot syndrome	3	0	0	0
Radiation dermatitis	4	0	1	0
Neuropathy	15	0	0	0
Proteinuria	2	0	0	0

### Efficacy

All patients received pelvic MRI for restaging after neoadjuvant therapy. According to RECIST criteria, four patients (8.9%) achieved CR, 26 (57.8%) achieved PR, and the other 15 (33.3%) remained SD. No patient developed PD. At restaging, all patients had evidence of clinical response to treatment, but seven (15.6%) did not proceed to surgery for various reasons, among whom four patients refused permanent colostomy, two achieved CR and the last one refused surgery for unknown reasons. The median time period from the last dose of bevacizumab to surgery was 11 weeks (range 6.9–13.8 weeks).

Patients underwent surgical resection at a median of 55 days (range 32–77 days) after completion of radiotherapy. Among 38 patients receiving curative surgery, 32 (84.2%) had sphincter-sparing surgery, of whom three had combined resection of other organs (one underwent partial vaginectomy, one underwent partial ileal resection, and one underwent combined hysterectomy) (Table 3). Creation of a temporary diverting ostomy was performed in 13 cases. The median operative time was 3.7 h (range 1.8–6.5 h), and the median blood loss was 50 mL (range 20–200 mL).

**Table 3:** Surgical procedures and pathological evaluation of 38 patients who underwent surgery

Surgical treatment	No. of patients (%)
Type of surgery	
Low anterior resection	32 (84.2)
Abdominoperineal resection	6 (15.8)
Pathological evaluation	
TRG grade	
TRG 0	15 (39.5)
TRG 1	8 (21.1)
TRG 2	12 (31.6)
TRG 3	3 (7.9)
Pathological stage	
ypT0N0	15 (39.5)
ypT1–2N0	11 (28.9)
ypT3N0	10 (26.3)
ypT0N1	1 (2.6)
ypT3N2	1 (2.6)

All patients who underwent surgical treatment had local complete R0 resection. Results of histopathological examination are presented in Table 3. Sixteen patients (42.1%) were reported as ypT0, 11 (28.9%) as ypT1–T2, and the remaining 11 (28.9%) as ypT3. Positive nodes were recorded in 2 (5.3%) patients. Overall, 35 (92.1%) of the 38 patients achieved clinical down-staging, with T down-staging in 33 patients (86.8%) and N down-staging in 33 (97.1%) of the 34 patients with imaging-detectable lymph nodes at presentation. 15 (39.5%) patients achieved pCR and eight (21.1%) patients had near-pCR.

### Surgical complications

Anastomotic leak occurred in eight (25.0%) of the 32 patients who had sphincter-preserving surgery, of whom four (12.5%) needed further surgical intervention, and the other four presented with mild symptoms, demonstrating that conservative treatment was sufficient. Rectovaginal fistula that required surgery was found in one (2.6%) patient 1 month after the original operation. Incomplete intestinal obstruction and pelvic abscess occurred in one (2.6%) patient. It is noteworthy that fewer anastomotic leak cases were observed in patients who underwent a temporary diverting ostomy than in those who did not (2/13 vs. 6/19). The two anastomotic leak cases in the group with prophylactic ostomy had mild symptoms and thus did not need surgical intervention. The median time period from the last dose of bevacizumab to surgery in patients with anastomotic leaks was 11.1 weeks (range 10.1–12.1 weeks).

All of the 38 patients who underwent surgery received a median number of five (range 0–6) cycles of adjuvant chemotherapy. Among the seven patients who did not undergo surgery, three continued with the provided adjuvant treatment regimen, and the other four refused any subsequent treatment for personal reasons.

### Three-year survival rates

**Fig. 1:**
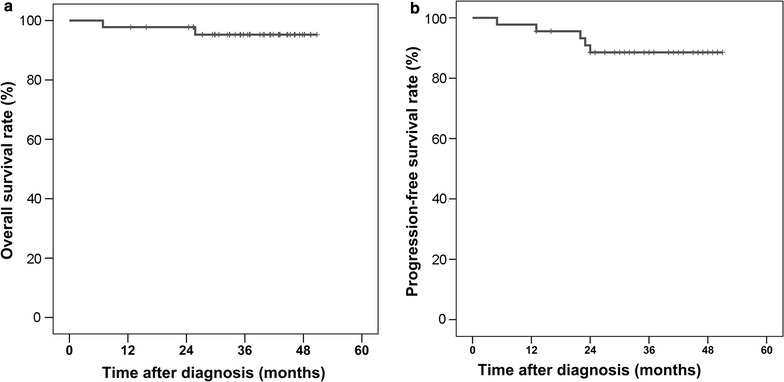
Kaplan–Meier survival curve of all patients enrolled in the present study. **a** Overall survival (OS) curve; **b** progression-free survival (PFS) curve

All patients were followed-up as scheduled over a median period of 37 months (range 7–50 months) (Fig. 1). To date, no participant who received radical surgery has had locoregional relapse. Only one died of lung and liver metastasis, and another developed para-aortic lymph node 14 months after surgery and continued with chemotherapy and radiation therapy. The 3-year PFS rate was 91.9%, and the 3-year OS rate was 97.1% for those who underwent surgery. For the seven patients who refused subsequent surgery, five remained SD, one died of peritoneal dissemination in the seventh month from diagnosis, and one experienced local progression and then continued with surgical resection of primary tumor and remained alive. Overall, the 3-year PFS rate was 88.6%, and the 3-year OS rate was 95.3% for the whole cohort (Fig. 1).

## Discussion

In this prospective phase II trial, we demonstrated that this multimodal neoadjuvant treatment for locally advanced rectal cancer, consisting of induction chemotherapy and concurrent chemoradiotherapy with capecitabine, oxaliplatin and bevacizumab, produced a satisfactory pCR rate and 3-year survival rate while slightly increasing adverse events and perioperative complications.

The most serious post-operative complication that surgeons were concerned about was anastomotic leak. The incidence of anastomotic leak that needed surgical intervention was 12.4%, slightly higher than studies without bevacizumab [28] and lower than other studies with bevacizumab [18, 29]. As a matter of fact, we did observe an increase in the incidence of anastomotic leak in the early stage of the study, so we suggested prophylactic ostomy as a standard procedure in the later stage. To our delight, the incidence of anastomotic leak was reduced after that and no further surgical intervention was needed. Although the importance of prophylactic ileostomy or colostomy on decreasing anastomotic leak has been widely verified in western countries [30], many patients and some surgeons in China do not seem to have accepted this idea. As a result, the beginning of this trial witnessed the creation of a prophylactic ostomy that was not mandatory but was determined by the surgeon and the patient instead. In fact, one anastomotic leak case that required surgical intervention was preventable, indicating that this patient did not refuse prophylactic ostomy. According to one study [31] in our hospital in 2015 that compared surgery with and without preoperative concurrent chemoradiotherapy for mid and low rectal cancer, the incidence of anastomotic leak in the concurrent chemoradiotherapy plus TME surgery group and the TME group were 2.2% and 8.5%, respectively (*P* = 0.101). Comparison of the incidence rate of leaks in concurrent chemoradiotherapy with the current study (2/90 vs. 2/13, *P* = 0.077) revealed a trend towards increased anastomotic leak, although no statistical significance was observed. According to the NCCN guidelines, at least a 6-week interval between the last dose of bevacizumab and surgery was recommended for the risk of healing complications. In our study, patients who developed anastomotic leak all had an interval longer than 10 weeks between the last dose of bevacizumab and surgery. Whether the anastomotic leak is related to bevacizumab remained unclear. Other toxicities were all acceptable. Hand-foot syndrome and oxaliplatin-related neurotoxicity were all mild, and no subsequent treatment was affected. Bevacizumab-related side effects such as hypertension and proteinuria were relatively few. Blood loss and operative time were comparable to patients who did not receive bevacizumab [31]. The incidence of adverse events in the present study was lower than in the literature. This may partly be explained by racial differences, because the acute adverse events that occurred during concurrent chemoradiotherapy in previous studies were also lower in Asian patients than in other ethnic backgrounds [20–22]. Moreover, as a study with small sample size, sample bias might also exist.

Bevacizumab was among the first molecular-targeted agents to be introduced to treat locally advanced rectal cancer when we began the present study in 2013. In preclinical models, bevacizumab has been found to lead to vascular normalization, reduce tumor hypoxia, improve radiosensitivity [32], and augment tumor response [15, 16]. All these effects have made bevacizumab a promising drug whose concurrent application with radiotherapy is worthy of being studied. Studies have also shown that adding bevacizumab to regimens for metastatic colorectal cancer has improved survival [13, 14]. However, in colon cancer stages II and III, the addition of bevacizumab to the systemic treatment has not shown any benefit. Therefore, whether bevacizumab has any advantage in treating micrometastases in locally advanced rectal cancer remains unclear. For better local and systemic control, researchers have attempted to integrate bevacizumab in the multimodal management of locally advanced rectal cancer patients, and several phase II studies have been conducted to test its safety and efficacy. The strategies developed by most studies integrated bevacizumab as a companion to conventional fluoropyrimidine-based [33–35] or intensified oxaliplatin-containing chemoradiotherapy [36, 37], or as an induction treatment before chemoradiotherapy [18].

Oxaliplatin, as the most promising drug in previous single-arm studies, did not improve surgical outcomes such as pCR rate, but increased toxicity in the Italian STAR-01 study [38] as well as the US NSABP R-04 trial [39]. However, a meta-analysis shows that the addition of oxaliplatin to fluoropyrimidine in neoadjuvant chemoradiotherapy could modestly increase the pCR rate and reduce the rate of intra-abdominal or peri-operative metastases, and that the addition of fluoropyrimidine did not result in more surgical complications or postoperative deaths within 60 days despite increased toxicity [40]. Induction of XELOX has also achieved more favorable compliance with less toxicity [10]. In our previous study, we also found that neoadjuvant sandwich treatment [20] with the XELOX regimen added to the conventional radiation is well tolerated. Such discrepancies might be attributed to differences in race as well as drug delivery. This finding was confirmed by our current study, in which we used bevacizumab plus the XELOX regimen as an induction treatment before radiotherapy and concurrent treatment during radiotherapy. Differing from the NSABP R-04 and STAR-01 trials in which oxaliplatin was given weekly during radiation therapy, the current study adopted a strategy of 3 weeks on the XELOX regimen with oxaliplatin given at one loading dose. This difference in the use of oxaliplatin may be beneficial for systemic control.

An intensified preoperative treatment might enhance downsizing and downstaging of the primary tumor, and thus increase the likelihood of achieving pCR. A trend to higher pCR rates was noted when more systemic agents were added to standard chemoradiation [41]. Previous phase III trials of capecitabine or 5-FU-based chemoradiation reported pCR rates being 11%–15% [5, 42]. When bevacizumab was added to conventional fluoropyrimidine-based chemoradiotherapy, pCR rates were increased to 7.5%–32% [33–35], and the addition of bevacizumab to intensified oxaliplatin-containing chemoradiotherapy further increased pCR rates to around 18–40% in several phase II studies [37, 41, 43]. Meanwhile, induction chemotherapy in combination with bevacizumab may also contribute to benefit the pCR rate. In the present study, bevacizumab was employed in both induction and concurrent treatments, and the current cohort showed tumor downstaging in most patients with an ypT0 rate of 42.1%, pCR rate of 39.5%, and near-pCR rate of 21.0%, which were almost the best results of previous phase II studies.

In the present study, a discrepancy between pCR and cCR was also observed. Only four patients (8.9%) achieved CR according to radiology, but the number of pCR cases turned out to be 15 upon pathological examination. This may be related to meticulous clinical practice, in which any sign of residual mass on MRI was considered to be a possible residual tumor. A previous study [44] reported that cCR was associated with pCR in approximately 30% of patients; and this is in accordance with our results.

Survival results were also promising in the present study, with a 3-year PFS rate of 88.6% and a 3-year OS rate of 95.3%, indicating effective control over both locoregional and distant diseases. Three explanations may be applicable. First, this intensified neoCRT treatment strategy further reduced local relapse, thus contributing to survival. Second, the 3-week XELOX regimen plus bevacizumab eradicated micrometastases, resulting in good distant control. Finally, racial differences might exist in patients’ compliance and clinical response. A previous study reported that 3-year disease-free survival could be used as a surrogate parameter for long-term survival in colorectal cancer [45], thus we have reason to believe that patients in this group will have satisfactory long-term survival.

Our study has some potential limitations that require special consideration. Its major weakness is that we did not make prophylactic ostomy a standard of care in the primary design, and the incidence of anastomotic leak was relatively high in the first stage. In the later stage, however, the incidence was reduced when prophylactic stoma was performed. Furthermore, although the pCR rate could be used as a surrogate endpoint for efficacy, longer follow-up is needed to evaluate the impact of bevacizumab on more important endpoints, such as DFS and OS.

## Conclusions

Preliminary results suggest that the combination regimen of bevacizumab and XELOX administered in neoadjuvant therapy with radiotherapy may increase the risk of anastomotic leak, and therefore was not considered as a regular recommendation for locally advanced rectal cancer. However, creating a prophylactic ostomy might help to reduce the incident of this adverse event. Current data presented as pCR rate and 3-year survival were satisfactory, indicating promising long-term survival. For patients at high risk with locally advanced rectal cancer, XELOX in combination with bevacizumab may be a potential treatment option.

## Abbreviations

XELOX: oxaliplatin and capecitabine
pCR: pathologic complete response
TME: total mesorectal excision
NeoCRT: neoadjuvant chemoradiation
DFS: disease-free survival
OS: overall survival
VEGF: vascular endothelial growth factor
VMAT: volumetric modulated arc therapy
CEA: carcinoembryonic antigen
CA19-9: carbohydrate antigen 19-9
ERUS: endorectal ultrasound
MRI: magnetic resonance imaging
CT: computed tomography
UICC: International Union against Cancer
RECIST: Response Evaluation Criteria in Solid Tumors
CR: complete response
PR: partial response
SD: stable disease
PD: progressive disease
AJCC: American Joint Committee on Cancer
TRG: tumor regression grade
NCICTC 3.0: National Cancer Institute Common Toxicity Criteria 3.0
PFS: progression-free survival
